# Lead Detection in a Gig-Lox TiO_2_ Sponge by X-ray Reflectivity

**DOI:** 10.3390/nano13081397

**Published:** 2023-04-18

**Authors:** Valentina Arena, Emanuele Smecca, Salvatore Valastro, Corrado Bongiorno, Giuseppe Fisicaro, Ioannis Deretzis, Carlo Spampinato, Giovanni Mannino, Sandro Dattilo, Andrea Antonino Scamporrino, Sabrina Carola Carroccio, Antonino La Magna, Alessandra Alberti

**Affiliations:** 1 CNR-IMM, Zona Industriale Strada VIII n.5, 95121 Catania, Italy; 2 Dipartimento Scienze Matematiche e Informatiche, Scienze Fisiche e Scienze della Terra, Università Degli Studi di Messina, Viale F. Stagno d’Alcontres 31, 98166 Messina, Italy; 3 CNR-IPCB, Via P. Gaifami 18, 95126 Catania, Italy

**Keywords:** titanium dioxide, gig-lox, lead, X-ray analysis, critical angle

## Abstract

The importance of lead analysis in environmental matrices becomes increasingly relevant due to the anthropogenic spread of toxic species in nature. Alongside the existing analytical methods to detect lead in a liquid environment, we propose a new dry approach for lead detection and measurement based on its capture from a liquid solution by a solid sponge and subsequent quantification based on X-ray analyses. The detection method exploits the relationship between the electronic density of the solid sponge, which depends on the captured lead, and the critical angle for total reflection of the X-rays. For this purpose, gig-lox TiO_2_ layers, grown by modified sputtering physical deposition, were implemented for their branched multi-porosity spongy structure that is ideal for capturing lead atoms or other metallic ionic species in a liquid environment. The gig-lox TiO_2_ layers grown on glass substrates were soaked into aqueous solutions containing different concentrations of Pb, dried after soaking, and finally probed through X-ray reflectivity analyses. It has been found that lead atoms are chemisorbed onto the many available surfaces within the gig-lox TiO_2_ sponge by establishing stable oxygen bonding. The infiltration of lead into the structure causes an increase in the overall electronic density of the layer and, thus, an increment of its critical angle. Based on the established linear relationship between the amount of lead adsorbed and the augmented critical angle, a standardized quantitative procedure to detect Pb is proposed. The method can be, in principle, applied to other capturing spongy oxides and toxic species.

## 1. Introduction

The awareness that certain human activities can adversely affect the environment makes it necessary to monitor environmental matrices such as air, water, and soil to detect any anomalies due to pollution [1]. The monitoring process is effective when it leads to an understanding of the sources of pollution and, as a result, how to limit it. The first step is to map the substances that may be hazardous to humans and the environment, such as heavy metals [2]. The World Health Organization has identified a ranking of the top 10 dangerous chemicals of public health concern, and one of the metals mentioned is lead [3]. Lead, if ingested or inhaled, can cause several illnesses. Therefore, the application of systems to capture it during, after, or even before its release into the environment is crucial to avoid damages in the case of its uncontrolled diffusion. A material that can be suitable for this purpose is titanium dioxide. TiO_2_ is one of the most popular nanomaterials that has numerous applications in medicine [4], the food industry [5], green energy [6], and many more. In the medical field, in particular, TiO_2_ can be used for more effective delivery of anticancer drugs [4]. Moreover, TiO_2_ can be used to extend the storage life of food and to avoid waste [5]. TiO_2_ is also used for photocatalysis under UV light irradiation to transform dangerous species into harmless elements to protect humans and the environment [7,8,9,10].

In the renewable energy field, TiO_2_ is applied to boost the performance of Perovskite Solar Cells (PSCs) [6]. In particular, TiO_2_ is used as an Electron Transporting Layer (ETL), allowing the achievement of high-efficiency values primarily thanks to the convenient band alignment of the anatase polymorphism with the perovskite material.

The production of TiO_2_ layers is feasible through techniques based on chemical methods, such as spin coating and doctor blading [11], or based on physical methods, such as sputtering [12]. Among the others, gig-lox sputtering deposition [13], based on the Grazing Incidence Geometry of the solid titanium source coupled with the Local Oxidation of the species at the sample side, represents a recent and innovative physical approach for the deposition of TiO_2_. This process allows growing, at room temperature and without solvents, spongy layers with interlinked mesopores and nanopores. This multi-scale porosity, gained along a bottom-up oxidation process of spatially separated TiO_2_ seeds, is a huge benefit for the infiltration capacity to capture species of different sizes and natures. On the other hand, TiO_2_ layers deposited by standard parallel plate geometries are more compact [14]. Infiltration by soaking with N-719 [15] molecules was successfully tested in gig-lox TiO_2_ layers. N-719 is a photoactive dye molecule whose diameter is comparable to the gig-lox nanopore size and, indeed, an octopus configuration can be achieved. 

Gig-lox application was also extended to Perovskites. Sanzaro et al. [16] demonstrated that MAPbI_3_ can be infiltrated into layers of gig-lox TiO_2_ with the empty volume decreasing from 42% to 18%. Lead atoms were found to decorate the surfaces of the sponge deeply into the gig-lox layer to reach the inner interface.

Gig-lox TiO_2_ has been recently implemented to capture Pb atoms to prevent its release into the environment from PSC devices [17]. The possibility of trapping lead into the gig-lox TiO_2_ layer has been demonstrated by achieving up to 94% of Pb sequestration after 4 h of dipping in aqueous solution of lead iodide (PbI_2_) using a 340 nm-thick layer.

To complement consolidated methods, such as the inductively coupled plasma mass spectrometry ICP-MS [18], that detect lead in liquid environments, we propose a new approach based on X-ray reflectometry that has been applied to a solid gig-lox sponge after lead sequestration in a liquid environment for ex-situ investigations in dry conditions. This technique is based on the change of the critical angle for total external reflection of the X-rays from a solid material and has been applied to quantify the lead captured by a 340 nm-thick TiO_2_ sponge with an area 1.25 × 1.25 cm^2^ immersed in an aqueous solution of 10 mL with increasing concentrations of Pb, from 0.4 to 6.4 ppm. The method quantifies the captured lead, and it could be extended to other capturing spongy oxides and toxic species.

## 2. Materials and Methods

Gig-lox TiO_2_ sputtering deposition. The instrument used for the deposition of gig-lox TiO_2_ layers is a customized DC-pulsed Magnetron Sputtering equipment. Produced by Kenosistec s.r.l., it consists of a Titanium target at an incident angle of 12.7° with respect to the sample surface, coupled with the local oxidation of the species at the substrate side. The deposition is carried out at room temperature, and it is always preceded by a pre-sputtering process to clean the target and eliminate residual oxide layers. The gasses introduced into the system are (1) oxygen, which is the reactive gas, with a flow rate of 2 sccm, and (2) argon, which is the carrier gas, with flow rate of 69 sccm. A rotation of 20 rpm is applied to the substrate during deposition to increase layer uniformity. The samples were deposited on glass with a power of 140 W, a voltage of 330 V, a current of 424 mA, and a deposition rate of 3.4 nm/min. The thickness of the implemented samples is 340 nm. 

Pb quantification in a liquid matrix by Inductively Coupled Plasma Mass Spectrometry (ICP-MS) analyses. The process for the lead adsorption in gig-lox TiO_2_ consists of the immersion of samples 1.25 × 1.25 cm^2^ made of porous material into aqueous solutions with different concentrations of PbI_2_ (Sigma–Aldrich, 99.99% purity) for a period of 96 h to reach the maximum Pb uptake. The aqueous solution used as solvent was 10 mL of deionized water with a pH of 5.8. After immersion, the gig-lox TiO_2_ samples are shaken at 200 rpm with a flat orbital shaker (IKA KS 260 basic). Pb concentrations were assessed with a Nexion 300X ICP/MS using the kinetic energy discrimination mode (KED) for interference suppression. Before analysis, the sampled solution was diluted, acidified with nitric acid, and added to the internal standards required for quantifying lead ions. Each process was repeated 3 times for higher accuracy, which was validated by comparing it with a standard reference material, SRM 1643f—Trace Elements in Water.

Pb quantification in a solid matrix by X-ray Reflectivity (XRR) analyses. The instrument used for XRR analyses is a D8Discover Bruker AXS diffractometer with a Cu-ka source, a Goebel mirror, a 0.1 mm slit at the primary beam, a 0.2 mm slit, and a scintillator as a detector. The critical angle for total external reflection was measured correspondingly to a penetration depth of ~20 nm in the pure TiO_2_ material. The penetration depth, also known as attenuation length or absorption length, corresponds to an intensity loss of the primary intensity by 1/e (~40%). The critical angle is a parameter related to the electron density of the material. The XRR profiles were acquired with a step size of 0.002° and a time per step of 2 s in the 2θ range 0.35–0.70°.

Density Functional Theory Calculations. All calculations have been carried out at density functional theory level using the BigDFT software [19,20]. The interface with water is modelled by the soft sphere implicit solvation [21,22,23,24]. Core electrons and exchange correlation are described by soft norm-conserving pseudo-potentials along with the Perdew Burke Ernzerhol functional as implemented in the Libxc library [25]. A six trilayers 4 × 1 supercell has been employed to model the anatase TiO_2_ (101) surface (288 atoms). We built the supercell starting from the optimized bulk lattice parameters. Atoms at the bottom (non-hydrated) trilayer of the slab were fixed at their crystal bulk coordinates. Surface boundary conditions have been set for all surface calculations. The wavelet basis functions were distributed on an adaptive uniform mesh with a resolution of hgrid: = hx = hy = hz = 0.40 Bohr for all calculations. All geometry optimizations have been carried out at Γ point.

STEM analyses. They were performed with a Cs-corrected JEOL ARM200C at 200 keV, equipped with 100 mm^2^ JEOL energy dispersive X-ray (EDX) detector. A HAADF detector was used to acquire STEM images in scanning mode.

## 3. Results and Discussion

We investigated and quantified the lead amount captured by a multi-scale-porosity gig-lox sponge in a liquid environment with the application of an ex-situ dry method based on the X-ray interaction with the solid matter. In particular, we exploited the relationship between the critical angle for total external reflection and the electronic density of the materials viewed by the X-ray probe. We thus collected the X-ray beam reflected by the gig-lox sponge close to the total external reflection condition. The resulting intensity profile is characterized by a critical angle, by a slope featuring the intensity reduction vs. the increasing incidence angle, and (where applicable) by interference fringes that depend on the electron density, roughness, and thickness of the analyzed layer, respectively. The introduction of lead into the TiO_2_ matrix causes a change in the electron density of the material, resulting in a critical angle shift. Interference fringes are not visible in our specific case due to the layer thickness that is above a threshold value of ~200 nm. All data hereafter discussed refers to a gig-lox sponge with a thickness of 340 nm, but similar results are achieved by changing the thickness in a range up to 1000 nm. We further noted that lead capturing is a process that takes place from the sample surface towards the inner part of the layer, with the saturation of sequestered lead achieved at high concentrations. The saturation level can be extended by increasing the layer thickness.

**Figure 1:**
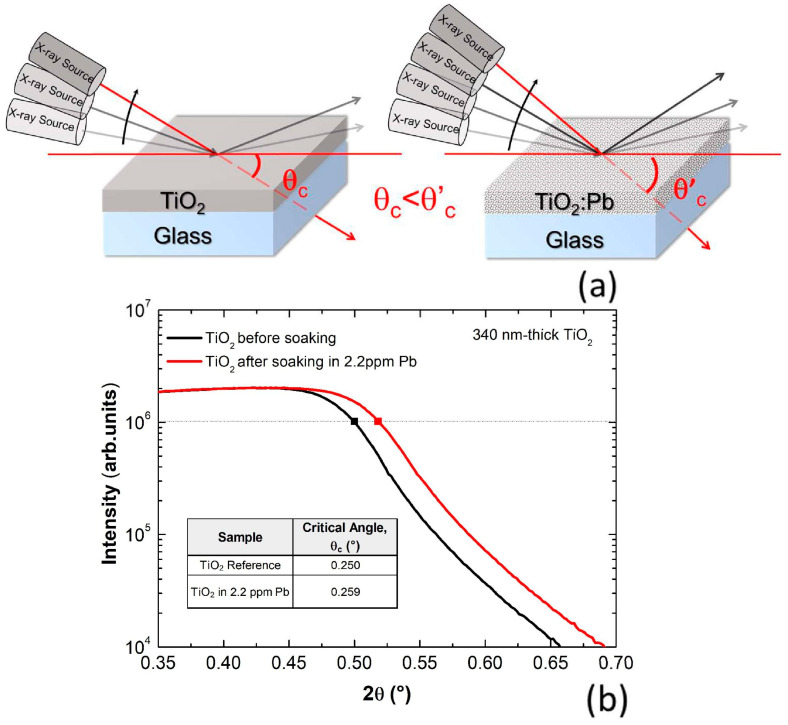
(**a**) Schematics of the working principle for X-ray reflectivity analyses on a gig-lox TiO_2_ sample deposited on a glass substrate. The presence of lead in the gig-lox films causes an increase of the critical angle θ_c_. (**b**) X-ray reflectivity curves from TiO_2_ films (340 nm-thick) before and after soaking into an aqueous solution containing 2.2 ppm of Pb. The black line represents the TiO_2_ sample before soaking and the red line represents the same sample after soaking, with its critical angle shifted rightwards.

Figure 1a shows a schematic of the XRR method applied to samples with different film densities, namely a pure gig-lox TiO_2_ and a gig-lox TiO_2_ containing Pb atoms. The schematic represents that the critical angle of the film with lead is higher than the one of a pure empty oxide due to the higher film density caused by the presence of Pb inside the film. To give an idea, the electronic density in lead is 2.6% higher than in a TiO_2_ moiety. Given the direct relationship between the square root of the electronic density and critical angle [26], an increase in θ_c_ occurs by lead incorporation into the TiO_2_ layer.

The XRR curves acquired before and after TiO_2_ soaking into a Pb aqueous solution at a concentration of 2.2 ppm of Pb for 96 h are shown in Figure 1b. The Pb capture and the consequent increase of the film density are translated into a shift of critical angle at higher 2θ values, as shown by the marked squares taken at half of the upper plateau, which univocally identifies the critical angle for total external reflection of the beam.

According to our results, a film of pure gig-lox TiO_2_ has a critical angle of θ_c_ = 0.250°. After immersion of the sample and consequent infiltration of the metal in the branches of gig-lox TiO_2_, the density of the film increases to θ_c_ = 0.259°.

**Figure 2:**
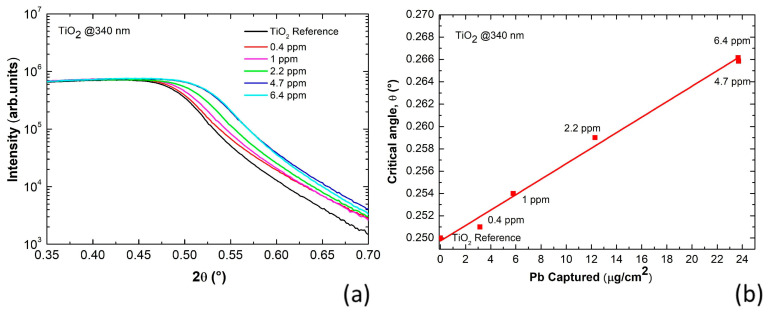
(**a**) XRR curves taken on gig-lox TiO_2_ layers soaked into solutions at increasing Pb concentrations. The progressively increasing amount of Pb captured by the sponge causes an increase in the θ_c_ values. A saturation is encountered at 4.7 ppm at the used sponge thickness (340 nm). (**b**) Critical angles as a function of the Pb captured by the sponge and measured by ICP-mass spectrometry. The linear fit has a slope of 6.93 × 10^−4^ (°cm^2^/μg) and an intercept of 0.25°.

The gig-lox TiO_2_ immersion was performed in five different solutions with increasing concentrations of Pb, from 0.4 to 6.4 ppm. We conducted the XRR measurements in all samples, including the pure TiO_2_ used as a reference, and the resulting curves are shown in Figure 2a. According to what is shown in Figure 1b, the critical angle monotonically increases by increasing the concentration of lead in the solution, from 0 to 6.4 ppm.

We measured the critical angles for all the samples as listed in Table 1. Those values were plotted as a function of the Pb amount that was captured by the film (Figure 2b) as experimentally measured by ICP-mass spectrometry. This method quantifies the residual amount of lead into the mother solution that has hosted the gig-lox layer. A high correlation between the captured lead quantity and the critical angle of the film was found, with a linear relationship established between them. This is further corroborated by a value of R-square equal to 0.99. The finding demonstrates that a progressively increasing amount of lead is captured by the sponge that is proportional to the concentration of Pb in the solution. Differently viewed, the curve in Figure 2b also represents a reference curve to quantitatively predict any amount of lead captured by the gig-lox layer based on the measured value of the critical angles. The mainstay of the method resides in the analyses that are done under dry conditions directly on the sponge instead of measuring the remaining lead amount in the mother solution after the immersion of the sponge. Our method can be easily extended to other porous oxides provided a reference curve is tailored. A universal curve over different materials requires specific evaluations and would be based on a similar integrated surface area available for lead capture with the same reactivity, and the same degree of pore filling that depends on the pore size and distribution.

**Figure 3:**
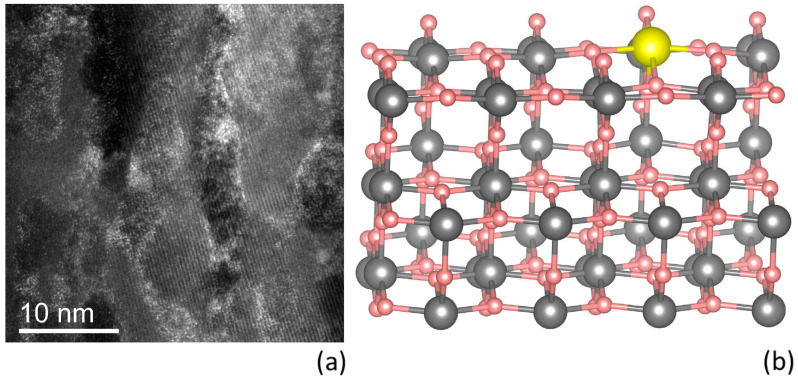
(**a**) STEM image of gig-lox TiO_2_ spongy layer after Pb sequestration. The distribution of Pb is uniform both in the meso and nano porosity of the gig-lox sponge. (**b**) Representation of one of the possible configurations of Pb bond with TiO_2_, wherein Pb is substitutional to Ti. In the figure: oxygen is pink; titanium is grey; lead is yellow.

To go deeper into the capture mechanism, we investigated the distribution of Pb atoms into the TiO_2_ branches. Figure 3a is a cross-sectional STEM image of the soaked gig-lox TiO_2_ layer at 6.4 ppm of Pb taken in the bottom part of the layer, characterized by its spongy ramification. Since the analytical method is sensitive to atomic mass contrast, lead is well visible in the image, even in very small amounts. The white spots are, in fact, Pb atoms. They are uniformly distributed over the porous layer and are well linked to the TiO_2_ surfaces. The number of lead atoms per surface unit was quantified as ~680 Pb atoms/nm^2^ for the entire thickness of 340 nm.

Different bonding configurations between Pb and the TiO_2_ layer could occur, especially considering that under-stochiometric surfaces are expected [16].

Density functional theory calculations have been employed to study the adsorption behaviour of a lead atom at the interior surfaces of the gig-lox TiO_2_ sponge, in contact with both vacuum and water. The gig-lox TiO_2_ is known to locally arrange in the anatase phase with main terminations along the (101) surfaces [13]. Previous calculations suggest that the anatase TiO_2_ (101) surface (named A_101_ hereafter) is the most stable among low-index surfaces [27]. As a consequence, we studied Pb adsorption at an A_101_ termination [24].

According to the preferential oxidation states of lead, equal to +2 and +4, and the relative electronegativity of the implicated elements, which decreases going from O to Pb and Ti, the Pb-O bonds are more stable than the Pb-Ti. At the perfect A_101_, both in contact with vacuum and water, a Pb atom forms three O-Pb bonds binding to three outermost oxygens. The adsorption energy of 3.40 eV at the A101/vacuum interface and 2.49 eV at the A101/water interface testifies to the intense interaction between lead and A_101_.

Although the synthesized TiO_2_ sponge is mainly characterized by (101) terminations [13], we expect that the interior cavities show a variety of superficial local atomic environments. As a consequence, the local superficial stoichiometry can vary from the perfect 1:2 ratio for Ti:O of the anatase TiO_2_. Pb adsorption on an A_101_ surface with an oxygen vacancy, with Pb replacing one of the eight nonequivalent oxygen atoms belonging to the first two O-Ti-O layers, is highly unstable with strong local atomic adjustments. In this case, when Pb replaces the outermost superficial site of oxygen, coordinated with only two Ti, is the more favorable adsorption configuration. Instead, the adsorption of Pb on an A_101_ surface with a deficit of titanium does not entail relevant rearrangements. Figure 3b shows the most stable configuration in vacuum among the four nonequivalent replacements of Pb with a Ti atom of the first two O-Ti-O layers. Differences in energy among the various oxygen adsorption sites are on the range of 1–2 eV, whereas the various titanium adsorption sites are below 1 eV, with such differences even below 100 meV in the case of the A_101_/water interface. Our study highlights a strong adsorption interaction of Pb both at the stoichiometric and defective termination in contact with vacuum and a water environment. Lead atoms can compensate and replace Ti sites in nonstoichiometric terminations with a deficit of Ti, preferring superficial sites lying at the vacuum or water interface of TiO_2_. The calculations prove the strong behavior of Pb atoms towards segregation on TiO_2_ surfaces where steady configurations with oxygen are obtained due to permanent loading during and after the diving in solutions.

**Figure 4:**
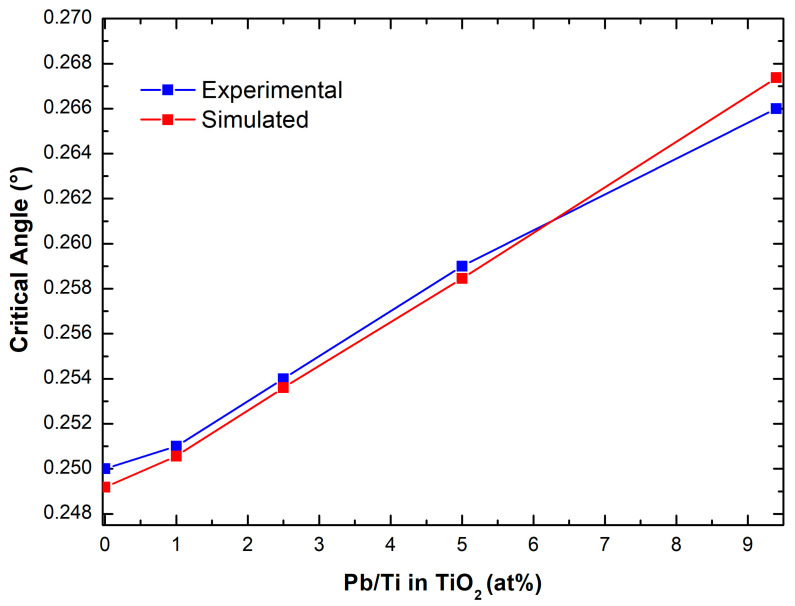
Raising of the critical angle of TiO_2_ for soaking at increasing lead concentrations in the solution: experimental and calculated values.

To further investigate the configurational arrangement of lead into the TiO_2_ sponge, the measured critical angles have been related to the measured Pb/Ti ratio, as shown in Figure 4. The sequence of points is almost linear, from the TiO_2_ reference sample to the maximum concentration of lead in the solution, which is 6.4 ppm. In the same graph, the measured critical angles are then compared to the values that were, instead, calculated by simulating a progressively higher Pb introduction into the gig-lox structure, as reported in Table 2. We considered a generic formula unit Pb_X_ Ti_(1−x)_O_2_**,** where x is the Pb index varying in the range 0-0.1, which represents the amount of Pb substitutional to Ti, as corroborated by the findings in Figure 3. An increase of x represents a progressive incorporation of Pb that is translated into an increasing electronic density of the overall material. The agreement between the two curves in Figure 4 supports the model to be applied to describe the process of lead capturing wherein the surfaces play a major role. We further observe that the experimental values of the critical angles are slightly and systematically above the simulated ones (except for the last point), representing that the pore filling must play another important role in the capturing procedure. We additionally calculated the critical angle by assuming a volume expansion of the TiO_2_ lattice in the range 0.1–1%. We found that a volume expansion under a threshold of 0.3% would be negligible on the calculation of the critical angle, supporting that the dominant effect is the lead atom introduction into the gig-lox structure.

## 4. Conclusions

In conclusion, we propose a new dry method to detect Pb soaked into a porous solid matrix,. The method is based on the measure of the critical angle for total reflection by an X-ray probe and it is applied to a gig-lox TiO_2_ sponge that has captured lead during immersion in a liquid solution. The method could be applied to other kind of sponges and toxic ionic species to be captured.

The gig-lox TiO_2_ sponge is a multi-porosity material made of interconnected meso (10–50 nm in diameter) and nano (1–5 nm in diameter) pores grown at room temperature by a modified sputtering process. The finding demonstrates that a progressively increasing amount of lead is captured by the sponge that is proportional to the concentration of Pb in the solution.

A calibration curve is shown to measure any amount of lead captured by the gig-lox layer based on the measured value of the critical angles for X-ray total reflection. Moreover, models and theoretical calculations to describe lead sequestration are proposed, indicating that the many available surfaces and their reactivity play a major role in establishing stable chemisorption of the pollutant species over the exposed surfaces through the sponge.

The method’s mainstay is the analyses, which are performed under dry conditions directly on the sponge rather than measuring the residual lead in the mother solution. The method paves the way for the quantification of pollutants in solid adsorbent matrices using X-ray analysis in the future.

## Figures and Tables

**Table 1:** Critical angle values for pure TiO_2_ and for each concentration of Pb in aqueous solutions.

Sample	Critical Angle, θ_c_ (°)
*w*/*o* lead	0.250
0.4 ppm	0.251
1 ppm	0.254
2.2 ppm	0.259
4.7 ppm	0.266
6.4 ppm	0.266

**Table 2:** Atomic percentage of Pb/Ti in Pb_X_ Ti_(1−x)_O_2_ with respective critical angles.

Pb/Ti in TiO_2_ (at%)	Simulated Critical Angle (°)	Experimental Critical Angle (°)
0	0.2492	0.2500
1	0.2506	0.2510
2.5	0.2536	0.2540
5	0.2585	0.2590
9.4	0.2667	0.2660

## Data Availability

The datasets generated during and/or analyzed during the current study are available from the corresponding author on request.
